# The Possible Protective Effect of Marital Status in Quality of Life Among Elders in a U.S.-Mexico Border City

**DOI:** 10.1007/s10597-017-0166-z

**Published:** 2017-09-08

**Authors:** Marisela Gutiérrez-Vega, Oscar Armando Esparza-Del Villar, Irene Concepción Carrillo-Saucedo, Priscila Montañez-Alvarado

**Affiliations:** Universidad Autónoma de Ciudad Juárez, Av. Plutarco Elías Calles 1210, Juárez, 32310 Chihuahua México

**Keywords:** Quality of life, Older adults, Marital status, Border

## Abstract

The purpose of this study was to determine how marital status may have an impact on quality of life in a group of older adults living in a U.S.-Mexico border city. Two-hundred and seventy-six older adults completed the Spanish version of the World Health Organization Quality of Life Assessment, composed of four domains: physical health, psychological health, social relationships, and environment. Participants answered a measure of sociodemographic variables. In the psychological health component of quality of life, single and married older adults had the highest scores as compared to widowed and divorced. Similarly, married older adults had the highest quality of life in social relationships. Marital status may play an important role when analyzing quality of life among older adults, this study suggests that being married may offer a protective mechanism against depressive symptoms and therefore against mental illnesses during late adulthood.

## Introduction

The first history records reported that children outnumbered their elders, however, in 2010 it was estimated that 524 million people were aged 65 years or older, that number represented the 8% of the world’s population (National Institute on Aging, n.d.). Life expectancy at birth has increased dramatically (Mathers et al. 2015), the causes of this phenomenon are reduction in mortality (Oeppen and Vaupel 2002), declines in fertility and improvements in longevity (National Institute on Aging, n.d.). During this century, the median age of the world’s population is projected to increase from 26 years in 2000 to 37 years in 2050 (Lutz et al. 2008). In Mexico, according to Gómez and Peña (2013), it was projected that the percentage of older adults in the world will be exceeded.

Due to the worldwide increase of the elderly population, it is important to examine the relationship between number of years lived and quality of life. A previous study evaluated the association between quality of life and number of years lived in 24 European countries. It was found a positive relationship between healthy life and quantity of years lived (Robine et al. 2009). However these results do not replicate in Latin American countries, particularly in Mexico. For instance, the Mexican elderly population suffers from many mental health conditions like depression, cognitive impairment, and dementia (Manrique-Espinoza et al. 2013). Also, research suggests that depression is the second cause of disability among the Mexican elderly population (Manrique-Espinoza et al. 2013).

The concept of quality of life is a multi-dimensional term that integrates objective and subjective indicators (Moons et al. 2006). The World Health Organization defines quality of life as:

The individuals’ perception of their position in life in the context of the culture and value systems in which they live and in relation to their goals, expectations, standards and concerns. It is a broad ranging concept affected in a complex way by the persons’ physical health, psychological state, level of independence, social relationships and their relationship to salient features of their environment (The World Health Organization Quality of Life Group 1995).

A research study analyzed the perception of quality of life and its association with socio-demographic, physical and contextual variables in a group of older adults (Fonseca et al. 2008). The sample was composed of 234 elderlies that came from a very low socioeconomic status (74%), most of them were married and living with their partner (63.7%). One of the instruments used in the study was the World Health Organization Quality of Life Instrument (WHOQOL-BREF), which contained 26 items evaluating the quality of their physical, psychological, social and environmental life. It was found that marital status was associated with physical, psychological and social quality of life. Overall, married couples had better quality of life in these components.

Gove et al. (1983) reported similar results in their study and concluded that married people had better mental health, widowed men had the poorest mental health, and divorced women had poorer mental health than divorced men.

Examining quality of life becomes significant and relevant when studying it in a U.S.-Mexico border town such as Juarez City. Juarez City was one of the border cities that registered the highest drug-related violence during 2008–2015 (Heinle et al. 2016, p. iv). Past studies have reported that violence is perceived as a public health problem because it causes physical damage, disability, and low quality of life (Hijar et al. 1997), thus quality of life is compromised when people experience violence (WHO 2002). The victimization process due to a social, political or economic factors can cause a considerable change in people’s lives (Gurrola et al. 2014). According to Echeburúa (2004), violence can be experienced directly when a person is attacked or assaulted, or indirectly when the person witnesses or hears about an aggression without being personally attacked. The vast majority of Juarez citizens experienced indirect violence when hearing brutal acts occurred in the neighborhood, gas station, or convenience store. People interrupted their routine activities, such as walking outside, going to restaurants, or if they had to go out there was always a feeling of uncertainty. The sequels of violence can be present in a person’s life for a long time, its manifestations are somatic indicators and poor mental health (Prince et al. 2007).

Studying quality of life in a violent environment, such as Juarez City, becomes especially relevant because there is no previous research on this matter. Besides, it is important to analyze the possible differences in quality of life due to marital status in an elderly sample. The goal of the study was to analyze the possible differences in quality in life among elders according to marital status, particularly after the period of high violence registered in the past years.

## Methods

### Participants

Participants were selected using non-probabilistic convenience sampling. The sample was composed of 276 older adults who were interviewed in different settings: waiting rooms at geriatric clinics, schools, health clinics, and recreational parks, all distributed throughout the entire city. All participants were living in Juarez, Mexico, at the time of the study. They were recruited during the time period of November 2014 to November 2015. The age range was 65–90 years; the mean age was 70.56 years (SD = 7.81). One-hundred and sixty-two were females and 114 were males. Only 43% of the sample had finished elementary school, 19% finished middle-school, 3% finished high-school, 3% finished college, and 32% did not attend school.

Forty-four percent (122) of the participants reported being married and living with their partner, 31.5% (87) were widowed, 11.2% (31) were single, 6.9% (19) were separated or divorced, and 6.2% (17) preferred not to answer. Most of the older adults reported a monthly income between $2,000 pesos and $3,000 pesos (between $110 and $165 dollars).

### Instruments

Participants answered the Spanish version of the World Health Organization Quality of Life Assessment (WHO 1996), the short form (WHOQOL-BREF) which contains 26 items. The Spanish version provides reliable and valid results since it has been used for evaluating many conditions (Lucas-Carrasco et al. 2011).

The items are grouped in four domains plus two global questions addressing overall quality of life and health satisfaction. The four domains are physical health (mobility, pain and discomfort, energy and fatigue); psychological (bodily image and appearance, negative and positive feelings, self-esteem, thinking, concentration); social relationships (personal relationships, social support, sexual activity); and environment (financial resources, freedom, physical safety and security, home environment). Greater scores on these domains reflect greater quality of life.

The internal consistency reliability measured by Cronbach’s alpha was 0.91 for the total scale. For each of the four domains that composed the entire scale, Cronbach’s alpha was ≥0.70; these results of internal consistency were similar to those reported by Lucas-Carrasco (2012).

## Results

The four groups of marital status (married, widowed, single, and divorced) were homogeneous with regard to education, χ^2^(12) = 17.88, *p* = 0.11 and sex χ^2^(3) = 2.33, *p* = 0.50. However, there were age differences among the four groups, *F*(3, 258) = 9.901, *p* < 0.001, post hoc analyses (Bonferroni) indicated a significant difference between single and married older adults (mean difference = 5.14, *p* < 0.01), and widowed and married older adults (mean difference = 5.19, *p* < 0.001); married older adults were the youngest, while single and widowed where the oldest with equal years of age. In the same way, differences with regard to socioeconomic status were evaluated. There were significant differences for socioeconomic status, *F*(3, 253) = 4.930, *p* < 0.01, post hoc analyses (Bonferroni) showed a difference between single and married older adults (mean difference = −1.06, *p* = 0.006); single and divorced older adults had the lowest level of income, while married and widowed had the highest level with similar incomes.

An Analysis of Covariance (ANCOVA) was performed in order to analyze the differences between marital status among the four different domains of quality of life while statistically controlling for possible effects of sex, age, and socioeconomic status.

There was no significant difference between marital status in the physical health domain, *F*(3, 254) = 2.60, *p* > 0.05. Even when the difference in physical health domain was not significant, single along with married older adults reported the highest scores, divorced older adults had the lowest score.

In the psychological component of quality of life, there was a statistical difference between groups, *F*(3, 254) = 2.99, *p* < 0.05. A post hoc Bonferroni test showed a significant difference between single and divorced older adults (mean difference = 13.67, SE = 4.71, *p* < 0.05), single older adults reported greater scores than divorced. Both single and married had similar scores, at the same time both groups had the highest scores, divorced older adults had the lowest score.

There was a significant difference between groups in the social relationships component of quality of life, *F*(3, 254) = 6.76, *p* < 0.001. A post hoc Bonferroni test showed that there was a significant difference between single and divorced older adults, (mean difference = 19.88, SE = 5.92, *p* < 0.01), single older adults reported greater scores. Also, there was a difference between married and divorced older adults (mean difference = 22.96, SE = 5.11, *p* < 0.001), married older adults had greater scores. And, there was a difference between widowed and divorced older adults (mean difference = 19.91, SE = 5.17, *p* < 0.01), widowed older adults reported greater scores. Among the four groups, married older adults had the highest score for social relationships, and divorced older adults had the lowest score.

There was no difference between groups in the environment component of quality of life, *F*(3, 254) = 1.88, *p* = 0.13. Even though the difference was not significant, married older adults reported greater scores along with single older adults, and divorced older adults scored the lowest on this domain.

## Discussion

The present study analyzed the possible differences in quality of life according to marital status among a group of elders. Even after statistically controlling for sex, age, and socioeconomic status, the results in this study showed that there is a difference in quality of life according to marital status, married older adults had greater quality of life in some of its components.

In the psychological health component of quality of life, single and married older adults had the highest scores as compared to widowed and divorced. It has been previously stated that in the Western world married people (both males and females) are more likely to report greater happiness than people who never married, got divorced or separated (Gove et al. 1983; Peters and Liefbroer 1997; Kahneman et al. 1999). Also, married people are more likely to enjoy a supportive and intimate relationship, thus are less likely to suffer loneliness (Kahneman et al. 1999). On the contrary, those who do not have a partner are less likely to share their emotions, everyday experiences, and thoughts. In the present study, those who reported being divorced, followed by widowed, had the highest frequency of negative feelings, helplessness, loneliness, sadness, and anxiety. These negative feelings deserve close attention since past research suggests that loneliness among the elderly is strongly associated with depression (Luanaigh and Lawlor 2008). Perhaps an accurate statement that has been previously reported is that “marriage is somewhat protective against loneliness” (Luanaigh and Lawlor 2008).

There is some research (Gove et al. 1983) that suggested that married men tend to score higher than married women in overall life satisfaction, mental health, and home life satisfaction. Thus, it may seem that married males benefit more than married females when talking about general mental health. In order to analyze these possible gender effects, in this study, it was decided to perform a 4 (single, married, divorced and widowed) x 2 (male/female) ANCOVA so that we could analyze a possible interaction between marital status and sex, however no gender effects nor interactions were observed, meaning that neither sex benefited more than the other. The fact that single older adults also had the highest psychological health along with married older adults deserves further investigation.

Related to the results in the psychological domain, married older adults had the highest quality of life in social relationships. The data showed that married older adults were more likely to report gratification from their friendships and people who surround them. These results are similar to those reported by Fonseca and colleagues (2008), who suggested married couples have better psychological and social quality of life.

Social support is believed to play an important role among older adults as it keeps them away from mental illnesses (Monahan and Hooker 1997). In a previous study it has been stated that having a reduced social relationship system was associated with higher levels of depression and suicidal ideation (Vanderhorst and McLaren 2005). Thus, people who have a strong social relationship system appear to have less depressive symptomatology than individuals who have a weak social relationship system (Stroebe et al. 1996).

The results of this study support the idea that marital status may play an important role when analyzing quality of life. Being in a relationship could be considered a “buffer mechanism” against psychological illnesses, reducing the likelihood of developing depressive symptoms, loneliness and isolation. This statement is particularly relevant when the findings come from a community that experienced social violence during the past decade, and that their quality of life could have been compromised.

### Strengths and Limitations

We believe this study is important since it is the first study that examines quality of life among the elderly community of a U.S.-Mexico border region. There was a previous descriptive study conducted in Juarez City (Gómez and Peña 2013) that examined the cognitive abilities of older adults, such as working memory, abstract reasoning, fluid and crystallized memory and dementia, but quality had not been assessed.

The relevance of this study lies in the importance of the construct of quality of life and how it can be easily compromised when the social environment does not ensure its existence. Thus, it is noteworthy that even in an environment of social violence, such as Juarez City, marital status could be considered a “protective mechanism” against social isolation, feelings of despair, anxiety, and depression, which many older adults may experience (Blazer 2003).

One limitation of this study was the number of participants, for future research it is intended to increase the sample size for ensuring representativeness. Also, it is important to mention that causality cannot be inferred from the present study. This is only one step towards better understanding of the possible influence of sociodemographic variables, such as marital status, in quality of life among the elderly in a border town that experienced violence for many years.

## Conclusions

This study evaluated the possible differences between marital status in quality of life. It was found that married older adults, along with single older adults, reported greater quality of life in the psychological domain. Also, married older adults had the highest scores in the social relationship quality of life domain. These results are consistent with those reported in past studies that stated that marital status is linked to positive mental health and quality of life. These results are particularly relevant for the U.S.-Mexico border region after a decade of social violence in which quality of life could have been jeopardized, for this vulnerable population marriage can be considered a protective mechanism.

The present study may also point out a specific elderly population that needs much attention in order to deal with feelings of loneliness, depression and suicide, particularly among those who are divorced and isolated. Local clinics, hospitals, government, public and private institutions should devote resources to create programs that promote quality of life from a comprehensive and focalized approach.
